# Inflammatory Cytokine Profiles of Semen Influence Cytokine Responses of Cervicovaginal Epithelial Cells

**DOI:** 10.3389/fimmu.2018.02721

**Published:** 2018-12-04

**Authors:** Cosnet L. Rametse, Anthonio O. Adefuye, Abraham J. Olivier, Lyle Curry, Hoyam Gamieldien, Wendy A. Burgers, David A. Lewis, Anna-Lise Williamson, Arieh A. Katz, Jo-Ann S. Passmore

**Affiliations:** ^1^Institute of Infectious Diseases and Molecular Medicine, University of Cape Town, Cape Town, South Africa; ^2^University of the Free State, Bloemfontein, South Africa; ^3^NRF-DST CAPRISA Centre of Excellence in HIV Prevention, Cape Town, South Africa; ^4^Western Sydney Sexual Health Centre, Western Sydney Local Health District, Parramatta, NSW, Australia; ^5^Marie Bashir Institute for Infectious Diseases and Biosecurity, Sydney Medical School-Westmead, University of Sydney, Sydney, NSW, Australia; ^6^National Health Laboratory Services, Johannesburg, South Africa; ^7^SAMRC-UCT Gynaecological Cancer Research Centre, University of Cape Town, Cape Town, South Africa

**Keywords:** genital inflammation, HIV, semen, cytokines, HeLa, interleukins

## Abstract

Genital inflammatory cytokine responses increase HIV risk. Since male partner semen is a complex mixture of immune-modulatory prostaglandins and cytokines, we hypothesized that exposure to semen may influence genital inflammation in women. Here, we investigated cytokine response kinetics of cervical cells following stimulation with seminal plasma from HIV-negative and HIV-positive men characterized as having low or high concentrations of inflammatory cytokines. Irrespective of the HIV status or semen cytokine profile, *in vitro* stimulation of cervical cells with seminal plasma resulted in significantly elevated concentrations of secreted IL-6, IL-8, TNF-β, MCP-1, GM-CSF, and VEGF within 8 h of stimulation, which tended to decline by 24 h, although this was only significant for TNF-β. Consistent with this, cervical cells responded to seminal plasma with increases in IL-8 and IL-1β mRNA expression of 10-fold. These findings suggest that the impact of semen on local female genital cytokines is likely transient. Although these findings suggest that the impact of semen on local female genital cytokines may not be sustained long-term, this heightened genital inflammation may have implications for HIV risk in women.

## Introduction

Semen is a complex fluid that ensures safe delivery of male genetic material into the female reproductive tract to ensure fertilization (1). In addition to ~15 million spermatozoa per milliliter of ejaculate, semen is a highly immune-modulatory mixture containing prostaglandin-E2 (PGE2), anti- and pro-inflammatory cytokines [including transforming growth factor (TGF)-β, interleukin (IL)-10, IL-8, macrophage colony stimulating factor (MCP)-1; regulated upon activation normal T cell expressed and secreted (RANTES), and secretory leukocyte protease (SLP)-1], all with the potential to alter the immune environment of the lower female reproductive tract (2–4). In addition to cytokines, seminal plasma also contains signaling molecules that induce expression of IL-1β, IL-6, and leukemia inhibitory factor (LIF) by endometrial epithelial cells (5, 6), which triggers recruitment and activation of macrophages, dendritic cells (DC) and neutrophils (6–10).

We have previously shown that >70% of HIV positive men had detectable HIV in their semen (3). The concentrations of inflammatory cytokines were generally similar in seminal plasma from HIV-positive and -negative men (3). In women, unprotected sex is associated with increased ectocervical macrophages, DCs and CD8^+^ T-cells, and decreased CD4^+^ T cells within 12 h following intercourse (6). Shortly after unprotected sex, TGF-β and vaginal pH were shown to be significantly elevated, while IL-8 and human beta defensin (HBD)-3 concentrations were lower (6, 10). These changes in cytokine responses in women were transient, however, with levels normalizing within 14 h. Similarly, ectocervical cell lines exposed to semen produced higher concentrations of IL-6, IL-8, IL-1α, and granulocyte macrophage colony stimulating factor (GM-CSF) (6).

Since inter-individual cytokine profiles of semen vary widely and according to HIV status (3), we investigated the temporal effects of seminal plasma from HIV-positive and -negative men with either high- or low cytokine profiles on *in vitro* cytokine responses by cervical epithelial cells.

## Materials and Methods

### Ethics Statement

Ethic approval obtained from the University of Cape Town Human Research Ethics Committee. Written informed consent was obtained from all men before sample collection.

### Male Cohort Recruitment

Thirty-eight HIV-positive and 28 HIV-negative men were enrolled from the Empilisweni Clinic in Athlone, South Africa (3). Ejaculates were collected in sterile specimen jars containing 6 ml of transport media [RPMI-1640 medium supplemented with 5 mM L-glutamine, 50 U/ml penicillin, 50 μg/ml streptomycin (GIBCO®, Invitrogen™, Carlsbad, CA, USA), 2 mg/ml fungin® (Invivogen, San Diego, CA, USA)] following voluntary self-masturbation. In the laboratory, semen was centrifuged at 1,000 × g for 10 min to remove cells and spermatozoa and seminal plasma was collected for subsequent experiments (3). All samples were processed in the laboratory within 2–4 h after donation and seminal plasma was stored at −80°C for viral load determination and cytokine measurements.

### STI Testing

A validated in-house real-time multiplex PCR assay was used to detect *Neisseria gonorrhoeae, Chlamydia trachomatis, Trichomonas vaginalis*, and *Mycoplasma genitalium* in DNA extracted from seminal plasma. DNA extraction was performed using the X-tractor gene platform (Qiagen, Germany) and M-PCRs were performed using Rotor Gene 3000 platform (Corbett Research, Australia) as described previously (11). The primers and probes used in the multiplex assay targeted *N. gonorrhoeae* cytosine-specific DNA methyltransferase gene, *C. trachomatis* cryptic plasmid, the *T. vaginalis* repeated DNA fragment, and *M. genitalium* pdhD gene, encoding for dihydrolipoamide dehydrogenase (12). After incubation at 50°C (2 min) and Taq activation at 95°C (10 min), 40 cycles of denaturation (95°C, 20 s), and annealing/extension (60°C, 60 s) took place. Genomic DNA extracts prepared from the following ATCC strains served as controls: *N. gonorrhoeae* (ATCC 700825), *C. trachomatis* (ATCC VR-885), *T. vaginalis* (ATCC 30001), and *M. genitalium* (ATCC 33530). Specimens that were positive for *C. trachomatis* were further tested for lymphogranuloma venereumassociated L1–3 serovars (12).

### Measurement of HIV Concentrations in Plasma and Semen

Plasma and seminal HIV-1 RNA concentrations (copies/milliliter) were quantified using NucliSENS EasyQ HIV-1 (version 2.0, bioMérieux SA, Lyon, France) according to the manufacturer's protocol. The assay had a lower limit of detection of 70 copies of HIV-1 RNA/mL and a linear range of detection up to 10 × 10^6^ copies of HIV-1 RNA/mL.

### Measurement of Cytokines in Seminal Plasma

The concentrations of 26 cytokines [including IL-1β, IL-2, IL-6, IL-7, IL-12p70, GM-CSF, IFN-γ, TNF-α, IL-1α, IL-8, IL-12p40, IL-15, eotaxin, fractalkine 3, G-CSF, MCP-1, MIP-1α, and−1β, RANTES, ITAC, monokine-induced by interferon Gamma (MIG), MIP-3α and TGF-β1, β2 and β3] were measured in semen using a human cytokine milliplex MAP kit (Millipore Corporation, St. Charles, Missouri, USA), according to the manufacturer's protocol. Seminal plasma samples were thawed and filtered by centrifugation using 0.2 μm cellulose acetate filters (Sigma, USA) prior to cytokine/chemokine measurements, to exclude debris (3). All values below the detection limit were recorded as half of the lowest measured concentration for each cytokine.

### HeLa Cell Culture and Stimulation

HeLa S3 cervical cells were cultured in Dulbeccos modified Eagles medium nutrient mixture F-12 (DMEM) with Glutamax-1 enriched with 10% FCS (Highveld Laboratories, Cape Town, S.A), including Penicillin (1,533 IU/mg) and Streptomycin (775 IU/μg) at 37°C and 5% CO_2_ (v/v). Cells were stimulated with a 1:50 dilution of seminal plasma in serum-free media for 8 and 24 h (13). Untreated cells were included as a negative control. HeLa cell supernatants were harvested for measurement of secreted cytokines while cells were harvested into 1 ml TRIzol^®;^ reagent (Invitrogen, US) for mRNA extraction. All experiments were done in triplicate. Supernatants and cells were centrifuged and stored at −80°C until further use.

### Measurement of Cytokine Responses by HeLa Cells

Following exposure of HeLa cells to seminal plasma, the concentrations of IL-1α, IL-1β, IL-6, IL-8, GRO, GM-CSF, VEGF, TNF-α, IP-10, and MCP-1 were measured in HeLa cell culture supernatants using the human cytokine milliplex MAP kit (Millipore Corporation, St. Charles, Missouri, USA), according to the manufacturer's protocol, as described previously (3). HeLa culture supernatants were filtered by centrifugation using 0.2 μm cellulose acetate filters (Sigma, USA) prior to cytokine/chemokine measurements, to exclude debris. All values below the detection limit were recorded as half of the lowest measured concentration for each cytokine. In addition, HeLa cell total RNA was extracted using TRIzol® reagent (Invitrogen, US) and complementary DNA (cDNA) was synthesized from 200 ng/μl RNA using MultiScribe™ Reverse Transcriptase (Applied Biosystems, US), following the manufacturer's protocol. Primer sets for target genes, including IL-1β, IL-6, IL-8, GM-CSF, MIP-3α, VEGF, and human cyclophilin A (included as a house keeping gene) were designed (Supplementary Table 1) and PCR reaction conditions optimized for each primer pair. Quantitative real time qPCR was performed in duplicates by using SensiMix SYBR® No-ROX on Eco™ Real time PCR system (Illumina™, US). Expression of mRNA for each of the target genes was normalized to the house-keeping gene human cyclophilin A and reported as relative expression.

### Statistical Analyses

Descriptive statistics were used to describe demographic characteristics. Fisher's exact test and Wilcoxon rank sums tests were used to compare categorical variables and continuous data between groups, respectively. To compare between control and experimental qPCR data at each time point, a Wilcoxon matched pairs (non-parametric) or a standard matched pairs *t*-tests (parametric) were performed to determine if there was a significant difference in inflammatory cytokine mRNA expression in control and seminal plasma-treated cells. A confidence interval of 95% (*p* < 0.05) was selected as a cut-off point. To assess the effect of seminal plasma on cytokine concentrations, a linear regression model was fitted to log-transformed cytokine concentrations. Pearsons' correlation was used to evaluate the relationship between cytokine mRNA induction and protein concentrations. Adjustment for multiple comparisons was conducted using the false discovery rate to reduce false positive results. Statistical analyses were performed using SAS version 9.3 (SAS Institute Inc., Cary) and Graphpad Prism version 5 (GraphPad Software Inc., US).

## Results

### Characteristics of the Cohort

Semen collected from 28 HIV-negative and 38 HIV-positive heterosexual South African men were used for this study (Table 1) (3). The male donors median age was 42 years (range 23–58), which did not differ significantly by HIV status, and the majority (93%) were circumcised. Of the 38 HIV-positive men, 12/38 (31.6%) were on HAART (HAART+). The median plasma viral load in HIV-positive, HAART negative men was 10200 RNA copies/ml (IQR 2250–40000 RNA copies/ml), significantly higher than the matching median seminal viral load of HIV-positive men on HAART (1389 RNA copies/ml; IQR undetectable-20060 RNA copies/ml). The majority of HIV positive HAART negative men were shedding HIV in semen (73.1%, 19/26) and the viral shedding into the semen was correlated with HIV concentration in blood (*p* = 0.04, Rho = 0.4). In addition, 4/28 (14.3%) HIV-negative and 9/38 (23.7%) HIV-positive men tested positive for STIs (*N. Gonorrhea, C. trichomatis, T. vaginalis*, and *M. genitalium*), which did not differ significantly by HIV status (*p* = 0.51 Fishers' exact test).

**Table 1:** Clinical characteristics of men included in this study.

**Characteristic**	**HIV-infected**	**HIV-uninfected**
Number	38	28
Age [years; median (IQR)]	39 (34–44)	44 (37–51)
CD4 count [cell/mm^3^; median (IQR)]	391 (278–507)	–
Number of men on HAART	12/38 (31.6%)
Plasma viral load [RNA copies/ml; median (IQR)]^1^	10200 (2250–40000)	–
Genital tract viral load [RNA copies/ml; median (IQR)]^1^	1389 (LDL^2^-20060)	–
Number of men with detectable HIV RNA in semen [N/Total (%)]	21/38 (55.3%)	–
Presence of STIs (%)^3^	9/38 (23.7%)	4/28 (14.3%)

1 *Including only the 26/38 HIV+ men not taking HAART*.

2 *LDL, less than detectable limit (70 HIV-1 RNA copies/ml)*.

3 *STIs included Neisseria Gonorrhea, Chlamydia trichomatis, Trichomonas vaginalis, and Mycoplasma genitalium*.

### Seminal Plasma Cytokine Profiles

We previously reported that inflammatory, adaptive and regulatory cytokines were present in higher concentrations in semen than in matching blood samples with MCP-1, IL-8, Fractalkine, GM-CSF, MIP-1β, IL-7, IL-15, and IL-10 being detected at significantly higher concentrations in seminal plasma than in blood (3). Cytokines present at the highest concentrations in seminal plasma were MCP-1 (medians ranging from 829 to 13,064 pg/ml), MIG (12,221 to 15,003 pg/ml), RANTES (67 to 2,448 pg/ml), and IL-8 (220 to 3,183 pg/ml) (data not shown). Compared to MIG and MCP-1, TGF-β was detected at 15- to 150-fold lower concentrations, with TGF-β3 being present at the highest concentrations and TGF-β2 at the lowest. As we have previously reported, seminal plasma cytokines were present in similar concentrations in HIV-infected and uninfected men (3).

### Selection of Seminal Plasma with High or Low Relative Cytokine Profiles

**Figure 1:**
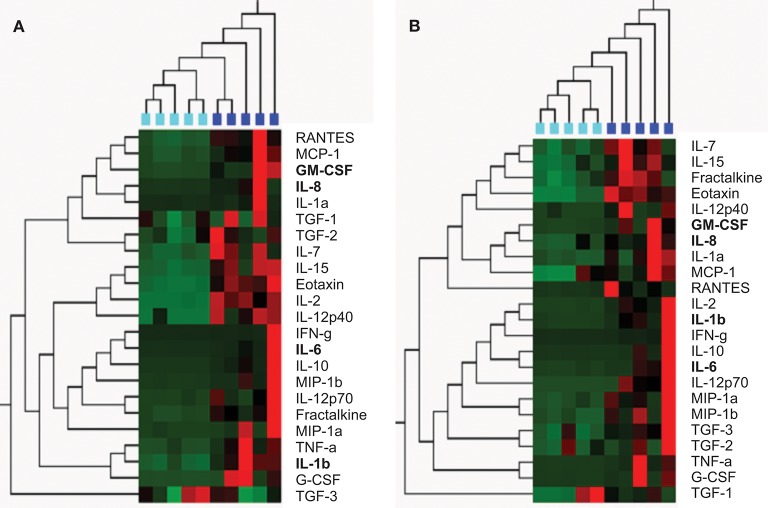
Unsupervised hierarchical clustering confirmed selection of semen from the HIV-negative **(A)** and HIV-positive **(B)** men by cytokine concentration; and to cluster men according to the similarities of their semen cytokine expression profiles (using Qlucore Omics Explorer). **(A)** HIV-negative men with the lowest concentrations of 23 cyokines (*n* = 5; turquoise blocks) clustered separately from HIV-negative men with the highest concentrations of these cytokines (*n* = 5; blue blocks). **(B)** HIV-positive men with the lowest concentrations of 23 cyokines (*n* = 5; turquoise blocks) clustered separately from HIV-positive men with the highest concentrations of these cytokines (*n* = 5; blue blocks). Cytokine concentrations are indicated using a color scale, ranging from green (low) to red (high). The dendrogram above the heat map illustrates degrees of relatedness between cytokine profiles evident within semen from the various men. In this tree, shorter and longer horizontal branch path lengths between pairs of cytokines, respectively reflect greater and lower degrees of similarity between the expression profiles of the assessed cytokines.Cytokines shown in bold indicate those that were measured in the *in vitro* experiments in this study.

From the 28 semen samples from HIV-negative men, five samples with the highest cumulative levels of inflammation (high inflammation) and five with the lowest cumulative levels of inflammation (low inflammation) were pooled for further analysis of the effects of semen and respective cytokine profiles on inflammatory responses by cervical Hela cells. Similarly, from the 35 semen samples from HIV-positive men, five samples with high relative cytokine concentrations and five with relatively low cytokine concentrations were pooled. To confirm that pooled seminal plasma were representative of high vs. low inflammation cytokine profiles, we performed an unsupervised hierarchical cluster analysis (Figure 1). Seminal plasma pools representing the highest relative cytokine concentrations and the lowest cytokine concentrations from HIV-negative and HIV-positive men clustered separately using unsupervised hierarchical clustering of relative cytokine levels. In contrast, seminal plasma pools representing the highest relative cytokine concentrations from HIV-negative and HIV-positive men clustered together and the same was observed for seminal plasma pools representing the lowest cytokine concentrations from HIV-negative and HIV-positive men. Table 2 provides a detailed summary of the panel of cytokines that were measured in the semen samples selected for each of these pools, similarly reflecting the fact that the low and high semen inflammation pools were significantly different for 18/26 cytokines measured for HIV± men and 19/26 cytokines measured for HIV- men. In semen, irrespective of HIV status, MIG and MCP-1 was detected at the highest concentrations while IL-12p70 were detected at the lowest concentrations.

**Table 2:** Cytokine concentrations in semen from HIV-infected and uninfected men with either high or low inflammation.

**Cytokine**	**HIV-infected [median pg/ml (IQR)]**		***P*-value**	**HIV uninfected [median pg/ml (IQR)]**		***P*-value**
	**High inflammation**	**Low inflammation**		**High inflammation**	**Low inflammation**
IL-1α	147.2 (61.5–616.7)	6.4 (4.9–11.0)	< 0.05	12.5 (6.6–793.0)	15.0 (1.7–39.3)	ns
IL-1β	9.1 (2.4–199.9)	0.02 (0.01–0.1)	< 0.05	1.0 (0.1–3.0)	0.3 (0.04–51.9)	< 0.01
IL-6	372.8 (0.7–1116.2)	7.9 (0.4–20.5)	< 0.01	92.7 (3.0–2635.2)	5.6 (1.3–164.2)	< 0.01
IL-8	3183.3 (119.2–9434.6)	270.1 (51.5–1771.8)	< 0.05	808.1 (161.4–18277.2)	220.4 (51.4–1841.4)	ns
MCP-1	13064.2 (2.3–28069.5)	829.0 (133.3–15539.6)	ns	14293.3 (2580.1–67443.7)	4077.0 (129.2–22424.9)	< 0.01
MIG	15003.5 (9598.9–34675.5)	12221.6 (3528.8–12221.6)	ns	13790.8 (9533.4–48812.6)	12637.3 (399–16802.6)	< 0.05
MIP-1α	249.5 (113.5–8658.7)	8.1 (1.3–19.1)	< 0.05	4.9 (1.3–508.5)	6.9 (1.3–25.0)	< 0.05
MIP-1β	262.3 (37.4–691.3)	21.1 (10.8–48.2)	< 0.01	97.5 (23.9–1899.7)	33.3 (15.6–196.3)	< 0.05
MIP-3α	6.2 (2.9–70.9)	0.4 (0.2–0.5)	ns	70.6 (7.1–630.1)	27.1 (2.0–161.8)	< 0.01
TNF-α	109.3 (64.9–1082.7)	18.8 (2.3–99.9)	< 0.01	6.6 (0.3–50.0)	2.1 (0.2–16.5)	< 0.05
RANTES	2448.0 (764.7–3502.8)	67.2 (40.6–117.2)	< 0.01	468.9 (72.5–1227.7)	114.1 (36.4–613.4)	< 0.01
IL-2	5.7 (2.7–23.8)	0.2 (0.02–0.3)	< 0.01	4.7 (0.02–8.3)	0.1 (0.02–7.3)	< 0.05
IL-7	2407.8 (1015.6–3216.0)	374.1 (145.6–575.2)	< 0.05	677.1 (134.1–3803.6)	258.4 (0.9–2667.4)	< 0.01
IL-10	53.9 (7.9–1066.3)	4.1 (0.7–4.9)	ns	166.6 (2.7–2959.9)	3.5 (1.6–294.2)	< 0.01
IL-12p40	25.7 (5.0–31.8)	1.3 (1.3–2.4)	< 0.05	2.6 (1.3–4.9)	1.9 (1.3–4.5)	ns
IL-12p70	2.0 (0.9–7.1)	0.02 (0.02–0.02)	< 0.05	0.02 (0.5–11.6)	0.2 (0.02–2.7)	< 0.05
IL-15	62.8 (34.7–1161.8)	8.1 (3.7–24.1)	< 0.01	30.9 (6.6–87.6)	7.6 (3.9–69.0)	< 0.01
IFN-γ	35.7 (17.2–552.1)	2.0 (0.9–2.3)	< 0.05	24.3 (0.3–1520.5)	1.5 (0.03–35)	< 0.05
G-CSF	59.9 (20.6–1700.3)	7.8 (1.7–11.2)	< 0.01	57.2 (7.9–1482.3)	15.8 (5.3–631.3)	< 0.01
GM-CSF	20.6 (3.7–73.5)	1.6 (0.4–1.9)	< 0.05	3.2 (0.5–48.9)	0.7 (0.03–9.2)	< 0.01
Eotaxin	38.1 (29.3–48.2)	2.4 (2.1–7.8)	< 0.05	44.1 (3.7–83.1)	5.2 (2.1–55.8)	< 0.05
Fractalkin	1085.1 (23.2–1567.0)	160.1 (63.0–351.1)	< 0.05	215.2 (152.1–5957.2)	234.8 (1.9–2068.6)	< 0.05
ITAC	1812.7 (833.1–1912.3)	414.6 (9.0–460.9)	ns	666.2 (269.7–1402.7)	689.5 (1.9–937.7)	ns

### Seminal Plasma Increased Cytokine Expression by Cervical Cells

**Figure 2:**
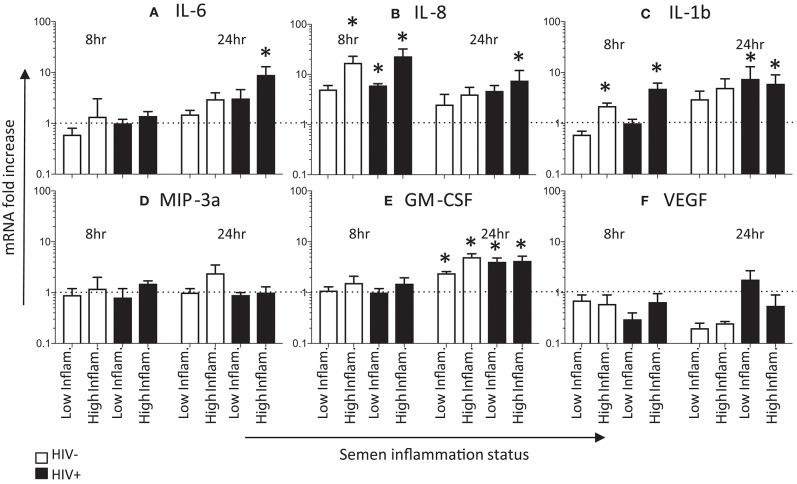
Effects of seminal plasma on cytokine mRNA expression by HeLa cells. Quantitative real-time PCR was used to determine the mRNA expression of **(C)** IL-6, **(A)** IL-8, **(B)** IL-1β, **(E)** GM-CSF, **(D)** MIP3α, and **(F)** VEGF by HeLa cells treated with 1:50 dilution of seminal plasma from HIV-negative (white bars) or HIV-positive individuals (black bars) for 8 h (*n* = 4) and 24 h (*n* = 3). The mRNA levels were normalized by housekeeping gene Human cyclophilin A. Data are represented as the mean ± SEM of duplicate wells in each experiment. Wilcoxon matched pairs (non-parametric) and standard matched pairs *t*-tests (parametric) were used to compare the control and treatment groups. *p < 0.05.

We compared cytokine mRNA expression after exposing HeLa cervical cells for 8 and 24 h with pooled seminal plasma from men with either low or high levels of inflammatory mediators (Figure 2). IL-8 mRNA expression was 5- to 10-fold increased following 8 h of exposure to semen with low- or high levels of inflammatory phenotype compared to mock exposed HeLa cells (*p* < 0.05 for HIV- high inflammation semen, and for both high- and low inflammation semen from HIV± semen), and occurred irrespective of HIV status of the semen donors (Figure 2B). IL-8 mRNA expression remained elevated at 24 h (significantly so for HIV+ high inflammation semen), although fold expression had decreased compared to the 8 h time point. IL-1β mRNA expression was elevated at 8 h in cervical cells exposed to seminal plasma with high (*p* < 0.05 for both HIV+ and HIV- semen) but not low levels of inflammatory molecules, which was similar in HIV positive and HIV negative seminal plasma pools (Figure 2C). IL-1β was further increased by 24 h, with mRNA being elevated in the presence of seminal plasma, regardless of the level of seminal plasma inflammation or HIV status. At the earlier time point, IL-6, MIP-3α, GM-CSF, and VEGF mRNA expression was not elevated compared to the control (Figures 2A,D–F). By 24 h, IL-6 mRNA expression was elevated in cells exposed to seminal plasma (Figure 2A), although this elevation was only significant for semen from HIV+ men with high levels of cytokines. GM-CSF (Figure 2E) expression was also only notably elevated at 24 h and not 8 h of exposure. While, VEGF and MIP-3a mRNA did not appear to be significantly elevated at either the earlier or later time point (Figures 2D,F).

**Figure 3:**
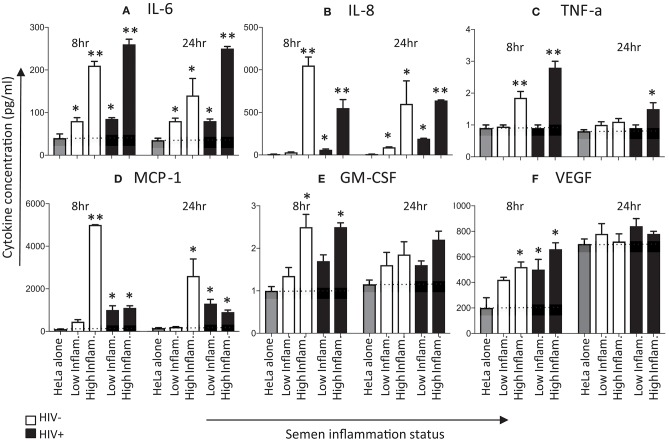
Effects of seminal plasma on cytokine secretion by HeLa cells. Hela cells were exposed to seminal plasma from HIV-negative (white bars) or HIV-infected individuals (black bars). A 1:50 dilution of seminal plasma from men with extreme inflammatory profiles (highest vs. lowest percentile) were used to stimulated HeLa cells for 8 and 24 h. Cytokine levels was measured in the 8 and 24 h post-stimulation in Hela cells supernatants by Luminex. Seminal plasma (1:50) in serum free culture media was used, while control was serum free culture media. Data are represented as the mean ± SEM of duplicate wells in each experiment. Wilcoxon matched pairs (non-parametric) and a standard matched pairs *t*-tests (parametric) were used to compare the control and treatment groups. *p < 0.05; **p < 0.01. **(A)** IL-6, **(B)** IL-8, **(C)** TNF-α, **(D)** MCP-1, **(E)** GM-CSF, and **(F)** VEGF.

Next, we determined cytokine production following exposure of HeLa cervical cells for 8 and 24 h with pooled seminal plasma (Figure 3). Protein concentrations were measured for four of the six cytokines for which mRNA (IL-6, IL-8, GM-CSF, and VEGF), in addition to TNF-α and MCP-1. Similar to the difference between seminal plasma with low vs. high inflammatory markers in IL-8 mRNA expression by HeLa cells, IL-8 protein concentrations were higher following stimulation with seminal plasma having higher concentrations of inflammatory cytokines in comparison to seminal plasma with lower concentrations of cytokines, irrespective of HIV status (Figure 3A). The seminal plasma with lower concentrations of inflammatory cytokines yielded significantly elevated IL-8 expression by 24 h (but not 8 h). While IL-6 mRNA expression was only evident at 24 h and was most evident when the cells were exposed to seminal plasma with high inflammation profile, IL-6 protein expression was significantly elevated at 8 and 24 h of exposure (Figure 3B). Seminal plasma with high inflammation profiles tended to induce more IL-6 protein expression than seminal plasma with low inflammation profiles, at both 8 and 24 h. GM-CSF and VEGF, both important growth factors that stimulate proliferation and maturation of lymphocytes and the formation of blood vessels, respectively, showed a similar profile of no to modest induction of mRNA (Figures 2E,F) associated with more clearly detectable induction of protein expression (Figures 3E,F). Protein expression of both of these growth factors appeared to peak early (8 h). While GM-CSF concentrations were higher after exposure to the high inflammation seminal plasma compared to the low inflammation seminal plasma, inflammatory status of seminal plasma did not appear to influence the induction of VEGF by HeLa cells. Comparing protein concentrations with mRNA induction for the four cytokines for which both measurements were performed, a moderate significant positive correlation was observed at 8 h (Pearson *R*^2^ = 0.2617; *p* = 0.04) but not at 24 h (Pearson *R*^2^ = −0.0961; *p* = 0.2).

TNF-α and MCP-1 concentrations tended to be induced early (Figures 3C,D), with TNF-α levels dropping to baseline by 24 h. The HIV status of semen donors did not appear to play a major role in induction of TNF-α, although MCP-1 concentrations appeared to be higher in the presence of seminal plasma from HIV-negative men with high levels of inflammation.

## Discussion

In addition to semen being a vehicle for spermatozoa delivery, it contains various immunological factors, including cytokines and chemokines that may influence the microenvironment within the female genital tract (2–4). This study focused on the effects of semen on inflammatory responses (mRNA and protein) *in vitro* by cervical epithelial cells. We found that semen resulted in significantly elevated secretion of IL-6, IL-8, TNF-α, MCP-1, GM-CSF, and VEGF within 8 h of stimulation, which declined by 24 h for the majority of cytokines. Irrespective of male HIV status or semen inflammatory profile, cervical cells responded to seminal plasma with increases in IL-8 and IL-1β mRNA expression of 10-fold after 8hr exposure.

Semen selected for this study was collected from HIV-negative and HIV-positive men with either low or high relative concentrations of 23 cytokines. As we have previously reported, MCP-1, MIG, RANTES, and IL-8 were all detected at high concentrations in semen, irrespective of HIV status (3). MCP-1 is a chemoattractant for monocytes, dendritic cells, and T cells (14, 15), MIG and RANTES are chemoattractants for T cells (16) while IL-8 is a potent chemokine that is involved in neutrophil chemotaxis (17). TGF-β, previously reported to be present in high concentrations in semen (10, 18–20), exists in different isoforms (TGF-β1, β2, β3, and β4) with close genetic relatedness, which plays an important role in normal growth and development (18–20), but is also central to many inflammatory processes at mucosal surfaces (10).

Both *in vitro* and *in vivo* studies have shown that female genital tract cervicovaginal cells exposed to seminal plasma have increased expression of GM-CSF, IL-6, IL-8, MCP-1, MIP-3α, IL-1α, and IL-1β (4, 6, 10, 21). This study was consistent with these previous findings, confirming the hypothesis that semen up-regulates pro-inflammatory gene expression in cells of the female genital tract. IL-8 in particular has been shown to promote HIV-1 infection (22) and the increase in IL-8 mRNA and protein expression following exposure to seminal plasma was found to be the greatest of all the cytokines evaluated in this study. IL-1β (together with TNF-α) has also been regarded as one of the main triggers of expression of pro-inflammatory cytokines and IL-1β mRNA was also one of the cytokine with the highest fold-induction in cervical cells following exposure to seminal plasma (23). This suggests that there is an inflammatory reaction that may be induced post-coitus during unprotected heterosexual intercourse, as shown *in vivo* (6).

To assess the biological significance of mRNA induction by HeLa cells following stimulation via seminal plasma, protein levels of IL-8, IL-6, GM-CSF, VEGF, TNF-α, and MCP-1 were measured. As with mRNA expression, cytokine concentrations (IL-6, IL-8, GM-CSF, VEGF, TNF-α, and MCP-1) were significantly increased in 8 h treated HeLa cell supernatants while only concentrations of IL-6, IL-8, GM-CSF, and VEGF remained significantly high after 24 h stimulation, irrespective of HIV and inflammatory status. Our findings confirm the hypothesis that seminal plasma up-regulates pro-inflammatory gene expression at both the mRNA and the protein levels. However, the cytokine mRNA induction correlated significantly with their protein levels only at 8 h but not at 24 h. This suggests that cytokine mRNA transcript abundances predicted cytokine protein concentrations only at the earlier time point. It was not clear why mRNA and protein levels correlated better at the earlier than later time point but this may be due to kinetic differences in mRNA expression and protein translation.

Although the effect of seminal plasma on the female genital epithelial tract has been measured *in vitro* (4), previous studies considered semen to be quite homogenous and have not considered the influential role of pro- and anti-inflammatory mediators present within seminal plasma on local female genital tract immune responses. The results from this study suggest that there is a relationship between the inflammatory cytokine content of seminal plasma and the extent of inflammatory cytokine mRNA expression they induce in Hela cells. This implies that men with higher cytokine levels in their seminal plasma may enhance inflammatory response in their female partners. Since this inflammatory response is characterized by cytokines that increase risk of HIV infection, this finding may suggest that males with high inflammatory profiles in their seminal plasma may put their female sexual partners at a higher risk for HIV infection.

From a survey performed on >1,000 men undergoing fertility testing, the mean volume of ejaculate produced was 3.8 ml (range 0.1–11.3) (24), which is a considerably higher concentration that the 1:50 dilution of seminal plasma we used in this and previous studies from our and other groups (25–27). Seminal fluid exerts no effect on HeLa cell viability at concentrations ≤ 1:50 (27). Since the aim of this study was to compare semen with high vs. low levels of inflammatory mediators on cervical cells, maintenance of HeLa cell viability was obviously a major consideration in selecting the concentration of seminal plasma to use. It is well-appreciated that this intrinsic toxicity of semen is related to the fact that post-ejaculation spermatozoon rapidly undergo apoptosis, via Fas-FasL interactions, apart from the few spermatozoa that successfully fertilize oocytes (28). Although we processed whole ejaculates from men within 4 h. of collection, it is possible that apoptosis of spermatozoa in the whole sample would have contributed to the toxicity potential of seminal plasma (27, 28), that would be independent of the inflammatory signals that semen plasma exert in the female reproductive tract which we were seeking to evaluate.

One of the limitations for using HeLa cells as a model for female genital cervicovaginal epithelial cell cytokine production is that these cells exhibit significantly different gene expression patterns from those in normal human tissues (29). While this means that extrapolation from the findings in this study to primary genital tract epithelial cells are not universal, the fact that this cell line is so well-characterized and homogenous in responses makes it a useful starting point for this type of study. Using three-dimensional genital cell cultures, like biopsy explant tissues (30), or organ-on-a-chip technology (31) could be considered in future studies. Another limitation of our study was that we did not measure sperm counts in the ejaculates, which may have influence their immune-modulatory potential. Von Wolff et al. (32) found that the immune-modulatory effect of seminal plasma was not associated with sperm counts. Furthermore, whole ejaculate samples were centrifuged immediately prior to semen supernatant removal, suggesting that the sperm component of the ejaculate was no longer present at the time the semen plasma was applied to the cervical cell line. We also did not exclude men with other STIs as these are major drivers of genital inflammation in men, and we considered it relevant to include infectious, as well as, non-infectious causes of inflammation in the semen donors that we selected.

In conclusion, we found that male seminal plasma up-regulates pro-inflammatory cytokine expression by cervical epithelial cells, and that the pattern of the cytokine concentrations in seminal plasma influences the extent to which this up-regulation occurs in HeLa cells. Furthermore, these findings suggest that the impact of semen on local female genital cytokines peaks relatively early following exposure and that changes may not be sustained long-term. This increased, albeit transient inflammatory response may elevate the risk of HIV acquisition in women during unprotected sex.

## Author Contributions

CR performed all the laboratory work, analyzed the data, and prepared the manuscript. J-AP conceptualized the study, supervised CR, analyzed the data, and co-wrote the manuscript. AO conceptualized the study, supervised CR, and helped prepare the manuscript. AA and AK supervised CR, assisted with some of the laboratory work, and contributed to the writing of the manuscript. WB conceptualized the male semen study and contributed to the writing of the manuscript. DL performed STI testing of male donors from which semen was derived, advised on data interpretation, and helped prepare the manuscript. LC analyzed the data and helped prepare the manuscript. A-LW provided funding and oversight for the parent study from which the male semen donors were derived, advised on data interpretation, and helped write the manuscript. HG supervised CR and helped prepare the manuscript.

### Conflict of Interest Statement

The authors declare that the research was conducted in the absence of any commercial or financial relationships that could be construed as a potential conflict of interest.
