# Supplementary material for: Inflammatory Cytokine Profiles of Semen Influence Cytokine Responses of Cervicovaginal Epithelial Cells
Source: Front Immunol. 2018 Dec 4;9:2721. doi: 10.3389/fimmu.2018.02721 (PMC6290331; doi:10.3389/fimmu.2018.02721)
Supplement: Supplementary file 1 [file Table_1.DOCX]

Supplementary Table 1 Primers used to measure mRNA expression by qPCR

| Gene Target | Primer | Sequence |
| --- | --- | --- |
| IL-6 | Forward primer  Reverse primer | GCCGCCCCACACAGACA  CCGTCGAGGATGTACCGAAT |
| IL-8 | Forward primer  Reverse primer | CTGGCCGTGGCTCTCTTG  TTAGCACTCCTTGGCAAAACTG |
| IL-1β | Forward primer  Reverse primer | TGTATGTGACTGCCCAAGATGAA  CTACCTGTGATGGTTTTGGGTATC |
| GM-CSF | Forward primer  Reverse primer | ATGTTTGACCTCCAGGAGCC  ATGTTTGACCTCCAGGAGCC |
| MIP-3α | Forward primer  Reverse primer | GGCGAATCAGAAGCAGCAAG  GACAAGTCCAGTGAGGCACA |
| VEGF | Forward primer  Reverse primer | ACATCTTCAAGCCATCCTGTGTG  CTCTCCTATGTGCTGGCCTTG |
| Human Cyclophilin A | Forward primer  Reverse primer | CCCACCGTGTTCTTCGACAT  CCAGTGTCTCAGAGCACGAAA |
